# Numerical Study of Electro-Osmotic Fluid Flow and Vortex Formation

**DOI:** 10.3390/mi10120796

**Published:** 2019-11-20

**Authors:** Wesley De Souza Bezerra, Antonio Castelo, Alexandre M. Afonso

**Affiliations:** 1 Faculdade de Ciências Exatas e Tecnologia, FACET, Universidade Federal da Grande Dourados, Cidade Universitaria-Rodovia Dourados-Itahum, Km 12, Dourados 79804-970, Brasil; 2 Departamento de Matemática Aplicada e Estatística, ICMC, Universidade de São Paulo, Av. Trabalhador São-carlense, 400, São Carlos 13566-590, Brasil; castelo@icmc.usp.br; 3 Faculdade de Engenharia da Universidade do Porto, Departamento de Engenharia Mecânica, Centro de Estudos de Fenómenos de Transporte (CEFT), 4200-465 Porto, Portugal

**Keywords:** HiG-Flow, HiG-Tree, finite differences, viscoelastic, electro-osmotic

## Abstract

The phenomenon of electro-osmosis was studied by performing numerical simulations on the flow between parallel walls and at the nozzle microchannels. In this work, we propose a numerical approximation to perform simulations of vortex formation which occur after the passage of the fluid through an abrupt contraction at the microchannel. The motion of the charges in the solution is described by the Poisson–Nernst–Planck equations and used the generalized finite differences to solve the numerical problem. First, solutions for electro-osmotic flow were obtained for the Phan–Thien/Thanner model in a parallel walls channel. Later simulations for electro-osmotic flow were performed in a nozzle. The formation of vortices near the contraction within the nozzle was verified by taking into account a flow perturbation model.

## 1. Introduction

For more than 200 years, it has been known that certain liquid solutions can flow through a small channel by applying an electrical potential difference at both ends of the microchannel. This area of study, or technique of manipulation of the fluid, i.e., the transport of particles through application of an external electric field, is called electrokinetics. In this scope, there are several applications in research such as chemistry, engineering and biomedical [1,2]. Electrohydrodynamics is the connection between theories of electromagnetism and hydrodynamics. Thus, the dynamics of fluids, electrokinetics and electrochemistry form the basis for the study of electrohydrodynamics. A general review and further details on the physical principles of electrokinetic effects can be found in [3].

One of the first studies of electro-osmosis is the work of Reuss [4], in which it is studied the flow of a polar fluid by application of an external electric field which acts on ionic solution, driving the movement of charged particles near the walls. At 1879, Helmholtz [5] reported the electrical flow parameters for the electrokinetic transport and he named of the Electrical Double layer (EDL). Subsequently, Goy [6] and Chapman [7] contributed in the studies of the flow distribution of charges near the walls in a capillary tube. In 1918 von Smoluchowski [8] performed capillary studies, generalizing the theory proposed by Helmholtz. Another great contribution was given by Debye and Hückel in 1923 [9]. They determined the ionic concentration in the solution by linearizing the Boltzmann distribution to the energy. Subsequently several studies related to electrokinetic effects in Newtonian fluids were performed. Later [10] carried out studies in ultra-fine capillaries, observing the effect of the surface potential on the flow. One year later Rice and Whitehead published the work on the surface potential effects for cylindrical capillaries [11]. Theoretical studies and numerical simulations of electro-osmotic flows began to be published by Yang and Li [12] and Patankar and Hu [13]. In 2000, Bianchi et. al. [14] solved the problem of electro-osmotic flow in a T-junction using the finite element method. Analytical solutions for Newtonian flow in channels were demonstrated by Dutta and Beskok [15]. In 2002 Lin et al. [16] solved numerically the Nernst-Planck equation for the ionic transport together the Navier–Stokes equations for Newtonian fluids. The study of electro-osmotic flow for non-Newtonian fluids is more recent, due to the difficulty imposed depending on the constitutive model to be used. In 2008 Park and Lee [17] determined the electro-osmotic velocity for viscoelastic flows while Zhao et al. [18] carried out studies of electro-osmotic flows of fluids modeled by the power law. Tang et al. [19] also performed a numerical study of non-Newtonian fluids in electro-osmotic flows. Afonso et al. [20] demonstrated the analytical solution for viscoelastic flows in a parallel plates and circular section channel, taking into account a non-zero pressure gradient and the electrokinetic effect, where one of the constitutive models used was the simplified Phan–Thien/Thanner (sPTT). In 2015, Peng et al. [21] studied the effects of a concentration gradient within a channel, taking into account a mixture of different electrolytic properties. Recently, Song et al. [22] carried out studies of numerical instabilities in mixing a ferrous solution with water in a parallel walls channel. Niu et al. [23] studied a microfluidic pumping by physical and numerical experiments in which it was found that the 2D flow obeys power law decay. In other study Niu et al. [24] demonstrate that self-generated solvent flow can be used to generate long-range attractions on the colloidal scale. The dynamics of this system is governed by an effective conservative energy that for large separations depends on the inverse of the distance. In this way, associated investigation of pumping including electrokinetic phenomenon were performed by [25,26]. Recently Arcos et al. [27], studied the behavior of a sPTT fluid subject to time dependent zeta potentials. Pimenta and Alves [28], study the implementation of electrically driven flow models of viscoelastic fluids in the finite-volume framework of OpenFOAM, applied the induced-charge electro-osmosis around a conducting cylinder and charge transport across an ion-selective membrane. Pimenta and Alves [28] code is freely available as open-source code.

Microfluidics have found widespread application in science and technology, as in medicine. The flow in microfluidic devices is usually created either by applying a pressure gradient (e.g., using a syringe pump), or using electrokinetic effects. Although effective for laboratory experiments, using a syringe pump is not practical for development of portable equipment for lab-on-a-chip applications, such as biological sample analysis. In contrast, electrokinetic effects are particularly useful at micro- and nanoscales. As an example, electro-osmosis can be efficiently employed to induce fluid pumping using electric fields, therefore avoiding the need to integrate micro-pumps and mechanical valves for flow control, which increase the complexity and the cost of disposable microfluidic devices. Small scales typical of microfluidics, increase the relevance of surface forces and electrokinetics effects and, for non-Newtonian fluids, there is an enhancement of the role of fluid elasticity, beyond anything that can be attained at macroscale. Thus, given the importance of the topic, in this work computational experiments were performed for electro-osmotic flows between parallel plates and in a nozzle. The vortices are introduced into the flow by supposing the existence of a perturbation near the corners in the channel contraction. The effect of viscoelasticity was also verified on the nozzle with the imposed electrical perturbation.

In Section 2, we describe the governing equations of the problem to the channels. Section 3 we present the computational method used to perform the simulations. Section 4 reports the verification for the parallel plates channel. Moreover, we present the proposed formulation for the nozzle flow problem and computational results obtained. Conclusions are drawn in Section 5.

## 2. Governing Equations

We are assuming the fluid incompressible laminar and isothermal flow. Moreover, here the treatment of the governing equations will be given in dimensionless form:

(1) ∇·u=0,

(2) $$\frac{\partial u}{\partial t}+u\cdot \nabla u=-\nabla p+\frac{1}{Re}\nabla^{2}u+\nabla \cdot S+F,$$

(3) $$T=\frac{2(1-\beta )}{Re}D+S,$$

where u is the velocity field, *t* is time, *p* is the pressure, $Re=\rho UH/\eta_{0}$ is the Reynolds number, *U* is the average velocity, *H* is the channel height, ρ the mass density and $\eta_{0}$ denotes the total shear viscosity $\eta_{0}=\eta_{s}+\eta_{p}$. The rate of deformation tensor $D=\frac{1}{2}\left(\nabla u+{\left(\nabla u\right)}^{T}\right)$ and T is the elastic stress. The dimensionless solvent viscosity coefficient is given by $\beta =\frac{\eta_{s}}{\eta_{0}}$. The evolution in time of the polymeric stress tensor is related by

(4) $$\frac{\partial T}{\partial t}+\left(u\cdot \nabla \right)T-\left[{\left(\nabla u\right)}^{T}\cdot T+T\cdot \nabla u\right]=\frac{1}{De}M\left(T\right),$$

where De=λU/H is the Deborah number and λ is the relaxation time of the fluid. Here we will be use a kernel-conformation tensor [29,30,31] and then determine the stress tensor. An alternative form to describe viscoelastic models is by using the conformation tensor, A. In general the equation for A can be written as

(5) $$\frac{\partial A}{\partial t}+\left(u\cdot \nabla \right)A-\left[A\nabla u+\nabla u^{T}A\right]=\frac{1}{De}M\left(A\right),$$

where M(A) is define according to the viscoelastic model. By taking into account the decomposition of the velocity gradient

(6) $$\nabla u^{T}=\Omega +B+{\mathrm{NA}}^{-1},$$

it was possible reformulate the tensor conformation, where in Equation (6) both Ω and N are anti-symmetric tensors, B is symmetric and commutes with A. This decomposition enables the rewriting of the constitutive equation for tensor A

(7) $$\frac{\partial A}{\partial t}+\left(u\cdot \nabla \right)A=\Omega A-A\Omega +2\mathrm{BA}+\frac{1}{De}M.$$

One of interest subject is to solve Equation (4) or Equation (5) for high values of Deborah number De. Numerical methods are unstable for certain critical values of De. In order to overcome such failure, Fattal and Kupferman [29] proposed a reformulation of the differential constitutive equations into an equation for the matrix-logarithm of the conformation tensor. Extending the ideas proposed by Fattal and Kupferman [29,30], Afonso et al. [31] presented a generic kernel-conformation tensor transformation that allows apply different kernel functions to the matrix transformation, in which the evolution equation for k(A), can be expressed in its tensorial formulations as

(8) $$\frac{\partial k(A)}{\partial t}+\left(u\cdot \nabla \right)k\left(A\right)=\Omega k\left(A\right)-k\left(A\right)\Omega +2B+\frac{1}{De}M,$$

where B and M are symmetric tensors constructed by the orthogonalization of the diagonal tensors. Thus, the HiG-Flow system solves Equation (8) instead of Equation (4), for more details please see [32]. Tensor A is only used in the HiG-Flow system to perform kernel-conformation, which is intrinsic to the system when viscoelastic flow simulations are taking out. In fact, numerical simulations in HiG-Flow can be performed using numeric stabilizers, where the kernel conformation is on, allowing the user to establish a stabilizer in a friendly manner without having to make modifications to the code kernel, but only in the input file of simulation. Considering a Newtonian fluid flow, the tensor S is null and the velocity and pressure are only updated at each step of time.

### 2.1. Phan–Thien/Tanner Model

Here we are interested in use the PTT model to solve the constitutive equation and then determine the velocity field. For this model, the right-hand side of Equation (4) can be written as:

(9) $$M\left(T\right)=\frac{2(1-\beta )}{Re}D-\left(1+\frac{\varepsilon \;Re\;De}{1-\beta }\mathrm{tr}\left(T\right)\right)T-\xi \;De\left(T\cdot D+D\cdot T\right).$$

The dimensionless parameter ε is related to the steady-state elongational viscosity in extensional flows and ξ is a parameter related with the molecular slip. If ξ is null, the model reduces to the simplified PTT (sPTT). On the other hand if ξ is not null, there will be a non-zero second normal-stress difference in shear, leading to secondary flows in ducts with non-circular cross-sections, which is superimposed on the streamwise flow [33]. In fact the right-hand side for the conformation tensor Equation (5) is given by

(10) $$M\left(A\right)=\left(1+\frac{\epsilon \;Re\;De}{1-\beta }\mathrm{tr}\left(S\right)\right)\left(I-A\right)-2\xi \;De\left(B-BA\right).$$

In this way the equations of motion can be solved for the PTT model fluid flow. Therefore, the electrical contribution must be taken into account for fully description of flow. In the next section we will show how the electrical source term was obtained.

### 2.2. Electro-Osmotic Force

Electro-osmotic fluid flows were first studied in the flow between a parallel wall microchannel and secondly in a contraction microchannel.

**Figure 1:**
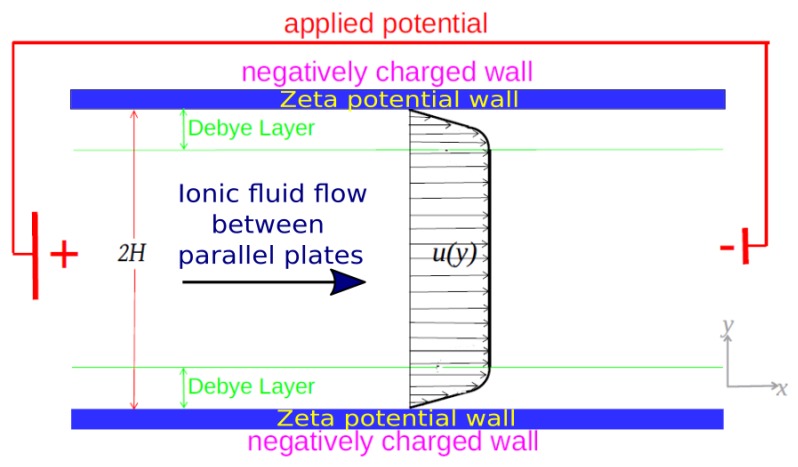
Scheme of electro-osmotic fluid flow in parallel plates. Velocity profile showed is fully developed for pure electro-osmotic fluid flow.

The schematic representation of electro-osmotic flow is show in Figure 1. The applied potential along the axis of the channel provides the driving force necessary to occur the electro-osmotic flow.

The PTT constitutive model [34] was used for studies involving electro-osmotic flow of non-Newtonian fluids. Numerical simulations are performed in two dimensions, taking into account L,w>>H, where *H* is the channel height, *L* is channel length and *w* the channel width. Moreover, due to the symmetry of the problem, we analyzed only half of the channel, i.e., 0≤y≤H.

For the problems subjected to the electro-osmotic forces, there exists a source term in Equation (2), $F=\rho_{e}E$, where E is the electric field. The electric field appears due to two contributions; one is the applied potential ϕ and the other due to the induced potential ψ which changes in the transversal direction to the channel walls. Thus, E=∇ϕ+∇ψ. The formation of the Debye layer occurs due to the spontaneous movement of the charged species near the channel wall, causing a charge redistribution in the fluid that originates the electrical double layer [35]. Therefore, the equations to be solved for these potentials are given by

(11) $$\begin{array}{cc} \nabla^{2}\phi & =0, \end{array}$$

(12) $$\begin{array}{cc} \nabla^{2}\psi & =-\rho_{e}, \end{array}$$

where $\rho_{e}=\delta \left(n^{+}-n^{-}\right)$ is the charge density, $\delta =n_{0}ezH^{2}/\epsilon \zeta_{0}$ with $n_{0}$ the reference concentration, *e* is the elementary charge, *z* the charge valence, ϵ the dielectric constant and $\zeta_{0}$ the potential on the wall. Moreover, the density charge is related by $n^{+}$ and $n^{-}$ which are the positive and negative ionic concentration, respectively. In this work we solve numerically the Nersnt–Planck equation for the ionic concentration:

(13) $$\frac{\partial n^{\pm }}{\partial t}=-u\cdot \nabla n^{\pm }+\frac{1}{Pe}\nabla^{2}n^{\pm }\pm \frac{1}{Pe}\alpha \nabla \cdot \left[n^{\pm }\nabla \left(\phi +\psi \right)\right],$$

where Pe=UH/D is the Peclet number that depends on ionic diffusion *D*. The potential to thermal energy ratio is given by $\alpha =ez\zeta_{0}/k_{B}T$ with $k_{B}$ the Boltzmann’s constant and *T* is the absolute temperature. The set of Equations (11)–(13) are solved to obtain the electrical force.

## 3. Computational Procedures

**Figure 2:**
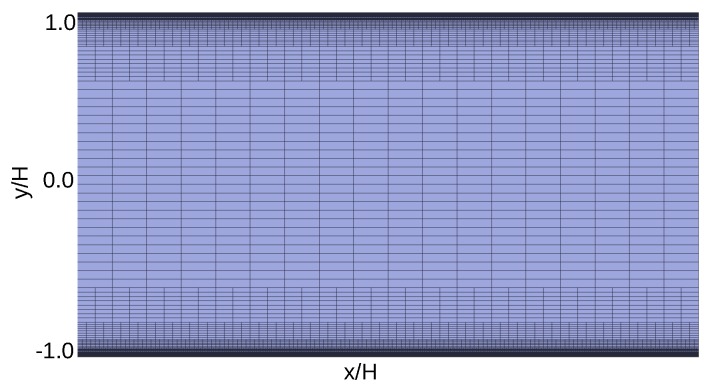
Computational grid used to perform the electro-osmotic fluid flow. The mesh is in scale. At the center of the grid Δx/H=0.2. This mesh has 8 levels of refinement.

**Figure 3:**
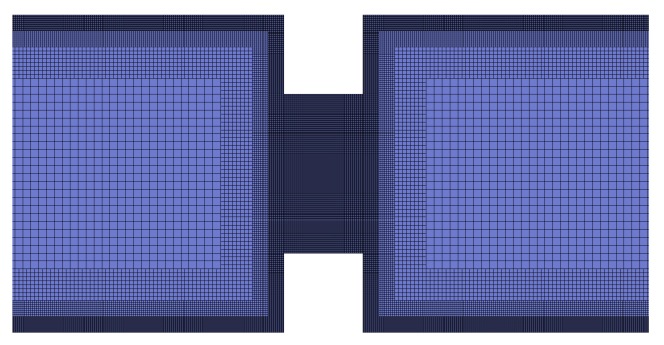
Illustration of the mesh used to the electro-osmotic simulations in a nozzle. This mesh has 4 levels of refinement.

The HiG-Flow system [32,36] was used to obtain numerical solutions reported in this work. Computational domain to the simulation is obtained through HiG-Tree, which generates a hierarchical mesh (for further information, refer to references [32,36]). For bi-dimensional case, this mesh is a generalized quad-tree [37]. Hierarchical meshes impose difficulties in the numerical scheme based on Cartesian approximations, and requires the use of spatial interpolations at unknown points of the stencil. The interpolations of the properties in the center of the faces and in the center of the cells are made by the technique of moving least squares, which uses a given set of points where the property is known to estimate a unknown value in a neighbor point. Differential equations are discretized by the method of finite differences. Solvers using the PETSc library (Portable, Extensible Toolkit for Scientific Computation) [38] are used to solve linear systems [32,36]. The machine used to perform numerical simulations has a Core i7 2.0 GHz CPU, 16 Gb memory. The mesh with refinements along the channel were obtained using HiG-Tree. The total length of the mesh is 20H. Better results were obtained with a maximum Δx/Δy=4. Near the walls the minimum size is $\Delta y_{min}/H=7.8125\times 10^{-4}$ and $\Delta x_{min}/H=3.1250\times 10^{-3}$. Figure 2 and Figure 3 shows the mesh used to simulate the electro-osmotic flow in parallel plates and in a nozzle, respectively.

**Figure 4:**
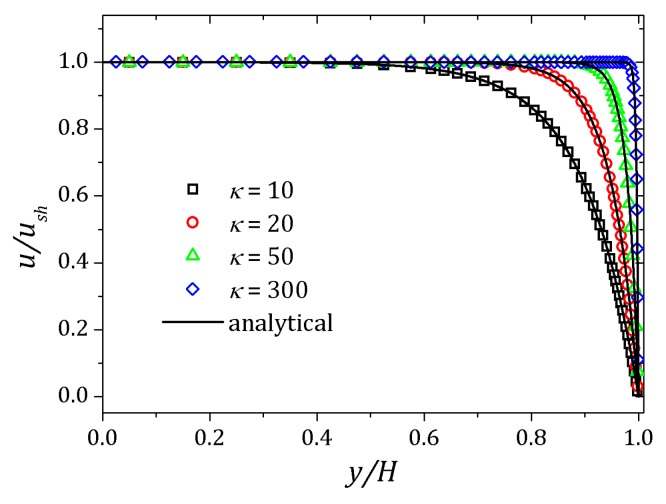
Velocity profile for Newtonian fluid flow. Results for κ=10,20,50,300.

**Figure 5:**
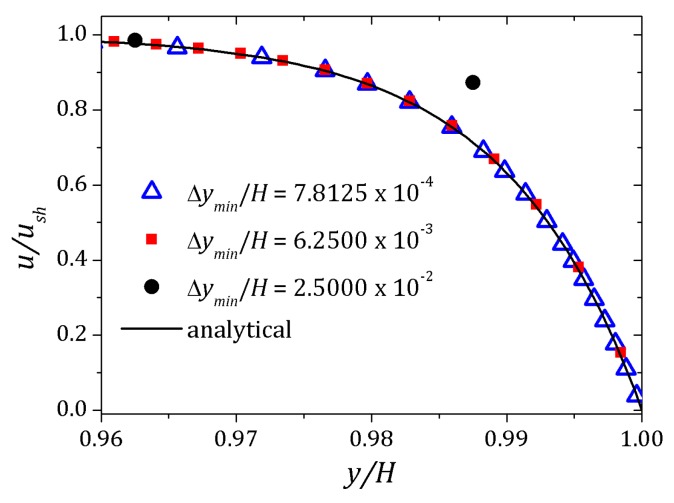
Effect of mesh refinement. Velocity profile of a Newtonian fluid subjected to electro-osmotic forces in a channel. We fixed κ=100 and vary the refinement near the wall. Image is zoomed near the wall to improved visualization of the refinement.

Figure 4 shows the velocity profile for different values of the Debye parameter defined as $\kappa =\sqrt{2\alpha \delta }$. As the Debye layer decreases, i.e., increasing the Debye parameter, variations in the velocity profile occurs near the channel wall. The curves are normalized by the Helmholtz-Smoluchowski velocity, $u_{sh}=-\epsilon \zeta_{0}E_{x}/\eta$. The dimensionless numbers $Re=1.0\times 10^{-3}$, Pe=1.0 were used. In order to illustrate the velocity profile convergence we were used three different grids. The main difference is the grid refinement, increasing the refinement near the walls to improve convergence. Effect of mesh refinement is show in Figure 5. Please note that for the mesh with $\Delta y_{min}/H=2.5000\times 10^{-2}$ represented by circles, the nearest point the wall is clearly outside the expected curve. Increasing refinement, $\Delta y_{min}/H=6.2500\times 10^{-3}$ represented by squares, all points remain within the analytical curve, but there are spaces with no points due the sharp effect near the wall and for $\Delta y_{min}/H=7.8125\times 10^{-4}$ represented by triangles, these spaces were filled and then the simulated curve agrees with analytical curve. In the next section we report the results.

### 3.1. Boundary Conditions on the Walls

On the channel walls, Neumann conditions apply for *p* and ϕ. The potential ψ assumes the potential reference value $\zeta_{0}$. The concentrations at the walls are given by the Boltzmann distribution Equation (17) and the non-slip condition is imposed for velocity.

(14) $$\begin{array}{cc} \hat{n}\cdot \nabla p & =0, \end{array}$$

(15) $$\begin{array}{cc} \hat{n}\cdot \nabla \phi & =0, \end{array}$$

(16) $$\begin{array}{cc} \psi & =\zeta_{0}, \end{array}$$

(17) $$\begin{array}{cc} n^{\pm } & =n_{0}\exp\left(\mp \alpha \zeta_{0}\right), \end{array}$$

(18) $$\begin{array}{cc} u & =0, \end{array}$$

### 3.2. Inflow and Outflow

Dirichlet conditions are applied for pressure, so the channel inlet and outlet have exactly the same pressure, resulting in a pure electro-osmotic flow, i.e., $p_{in}$ equal to $p_{out}$. Similar conditions were applied for the electrostatic potential ϕ, i.e., $\phi_{in}\neq \phi_{out}$ agreeing with ∇ϕ≠0. Concentration assumes the $n_{0}$ reference value at inlet, and the outlet Neumann condition $\hat{n}\cdot \nabla n^{\pm }=0$ is imposed to obtain a fully developed flow. It was taking Neumann condition for the velocity at the inlet and outlet; thus, the flow is developed under the action of the electric body force.

## 4. Results

### 4.1. PTT Model

The PTT model was used to solve the pure electro-osmotic fluid flow through parallel plates. We will first show the results potential and ionic concentration which are coupled properties of the flow, according to set of Poisson–Nernst–Planck equations given by Equations (11)–(13).

**Figure 6:**
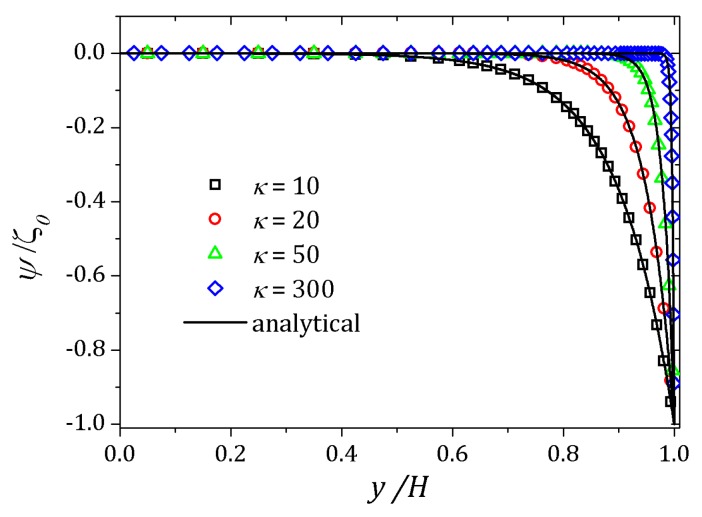
Potential ψ for different values of the Debye parameter. Results for κ=10,20,50,300.

**Figure 7:**
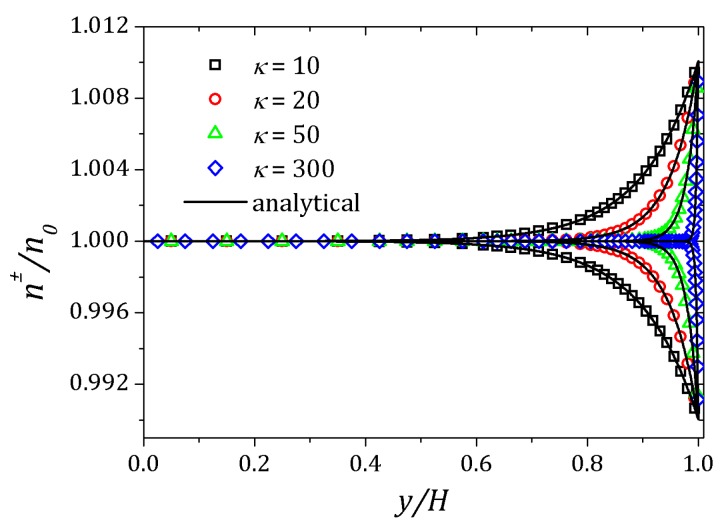
Ionic concentration $n^{\pm }$ for different values of the Debye parameter. Results for κ=10,20,50,300.

**Figure 8:**
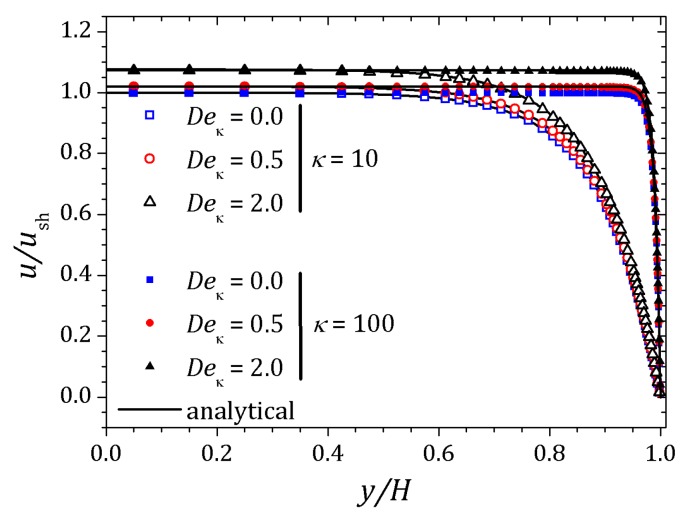
Velocity profiles to the full PTT model for κ=10 and 100. Results for ε=ξ=0.01, varying the Deborah number $De_{\kappa }=0,0.5$ e 2.0.

**Figure 9:**
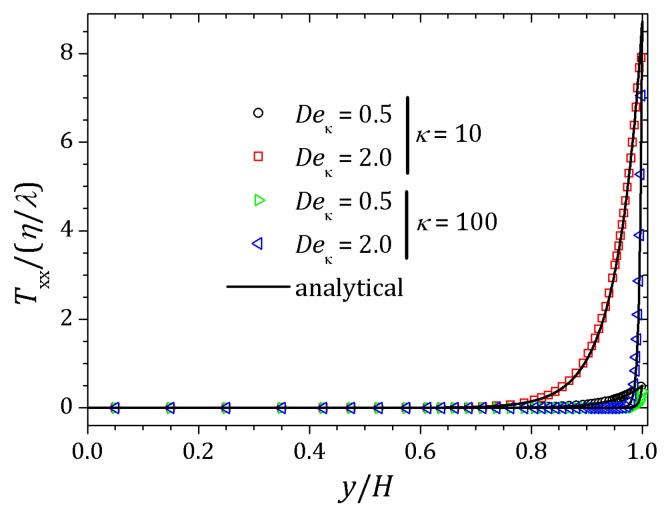
Normal stress for full PTT model. Results for κ=10 e 100 and fixed parameters ε=ξ=0.01.

**Figure 10:**
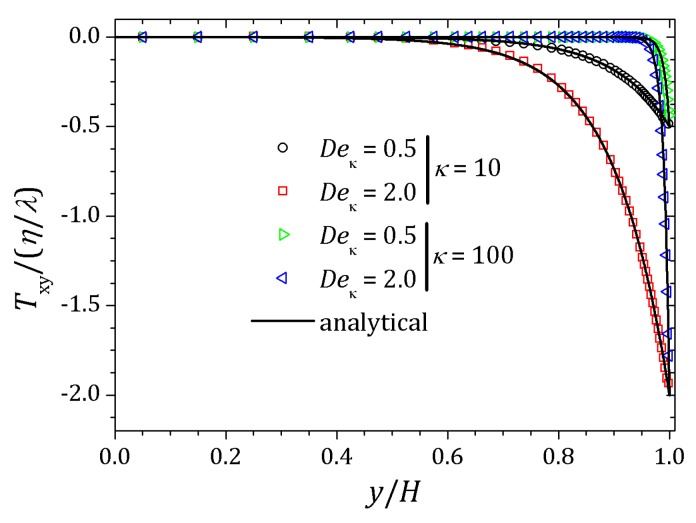
Shear stress for full PTT model. Results for κ=10 e 100 and fixed parameters ε=ξ=0.01.

Figure 6 and Figure 7 show the behavior of ψ and $n^{+}$, $n^{-}$ respectively. The distribution of charged particles causes the potential variation near the wall, and the ionic concentration near the wall is related by changes on potential ψ in that neighborhood. For κ=10 potential changes are seen at greater distances to the wall if compared to the curve show for κ=20. As the Debye layer becomes thinner, κ=50, the potential changes quickly near the channel walls so the charges are approaching to the walls as shown for κ=300. Therefor the velocity depends on *y* and κ it will also vary according to the relaxation time of the fluid. In this way, the velocity profile is affected by the Deborah number $De_{\kappa }=\lambda \kappa u_{sh}$ [20,39], as shown in Figure 8. For this problem we set $De_{\kappa }=0.5$ and $De_{\kappa }=2.0$. In addition, the parameters ξ=ε=0.01 were fixed. The Debye parameter assumed two different values, κ=10 and κ=100. For low Deborah number $De_{\kappa }=0.5$, the velocity profile indicates behavior similar to that of a Newtonian fluid. Increasing the number of Deborah is apparent the changes on velocity profile respecting to the Newtonian profile due to the low viscosities near the wall, in fact influenced by the appearance of the shear stress of the PTT model. The normal and shear stress are shown in Figure 9 and Figure 10, in which we can observe that the numerical results are similar to the analytical solution.

### 4.2. sPTT Model

In this model the parameter ξ=0 at the expression Equation (10). Moreover, instead of considering the pure electro-osmotic flow, we will impose a non-zero pressure gradient on the channel. In this sense, by taking into account the steady-state flow, the solutions for the velocity and the viscoelastic tensor for the sPTT model depend on the parameter Γ defined as:

(19) $$\Gamma =\frac{H^{2}}{\epsilon \zeta_{0}\;E_{x}}\frac{dp}{dx}.$$

**Figure 11:**
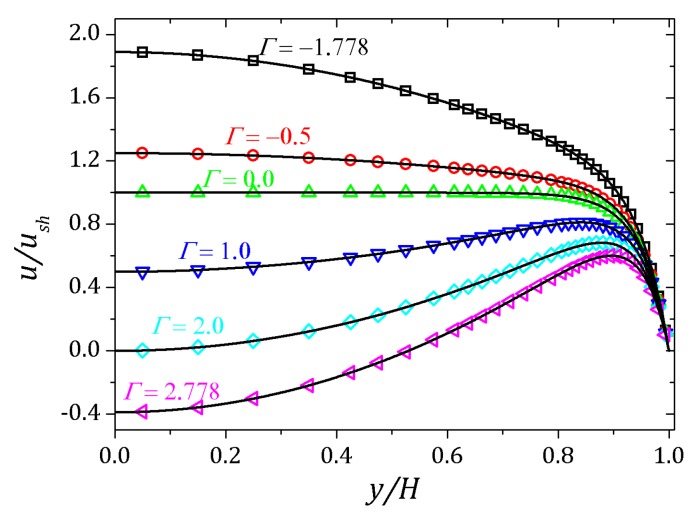
Velocity profiles for a Newtonian flow subject to an external pressure gradient. Results for κ=20.

If the pressure gradient is greater than zero, dp/dx>0, the fluid is pushed in the opposite direction of flow as shown in Figure 11 for Γ=1.0, Γ=2.0 and Γ=2.778, represented by downward triangles, diamonds and left-pointer triangles respectively. On the other hand, if dp/dx<0, the fluid is pushed in the same direction of flow, resulting in the profiles shown in Figure 11 for Γ=−0.5 e Γ=−1.778 represented by circles and squares respectively.

**Figure 12:**
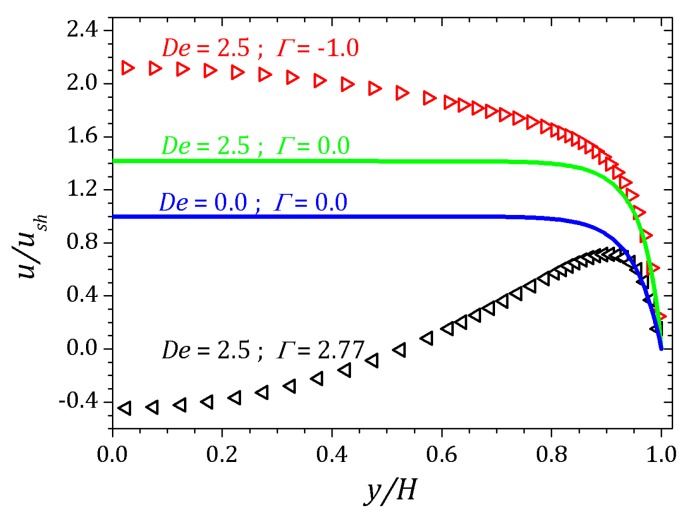
Velocity profiles for a sPTT fluid flow subject to an external pressure gradient. Results for ε=0.1, κ=20.

The Figure 12 shows the velocity profile of the electro-osmotic flow for sPTT fluid. For Γ=−1.0 represented by right-pointer triangles and Γ=2.77 by left-pointer triangles. As with the full PTT fluid, the velocity profile in the sPTT model is influenced by the Deborah number, which in these simulations is $De_{\kappa }=2.5$. In fact there exists a dependence on $\epsilon De_{\kappa }^{2}$. More details, please see [20].

**Figure 13:**
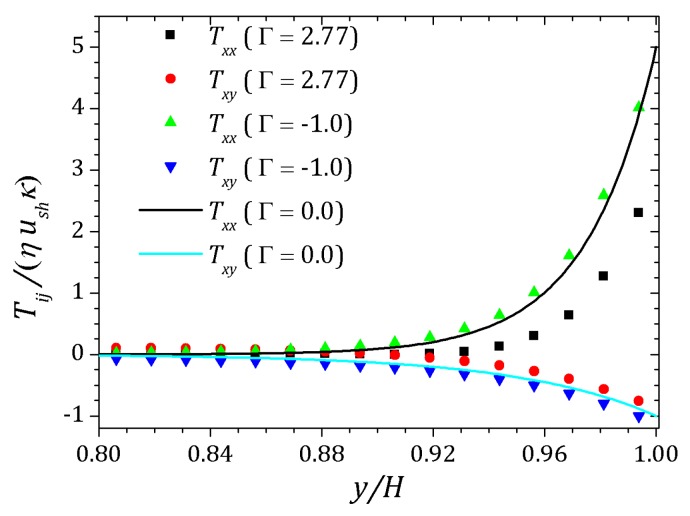
Effect of application of the pressure gradient on stress tensor. Normal and shear stress for $De_{\kappa }=2.5$, ε=0.1 e κ=20.

This effect is understand for the curves when Γ=0 showed in the Figure 12 represented by solid lines for $De_{\kappa }=0$ and $De_{\kappa }=2.5$, where it seen changes relative to the Newtonian profile. Normal and shear stress are shifted relative to the corresponding curves for pure electro-osmotic fluid flow which Γ=0 represented by solid lines as shown in Figure 13. For the normal component $T_{xx}$, the curves indicate the decrease of the stress near the channel wall when the pressure gradient is positive, Γ=2.77, represented by upwards triangles. On the other hand, if the pressure gradient is negative, Γ=−1.0, so $T_{xx}$ increases near the wall, as can be seen the curve represented by downwards triangles. Similar behavior is observed for shear stress $T_{xy}$.

### 4.3. Fluid Flow in a Nozzle

**Figure 14:**
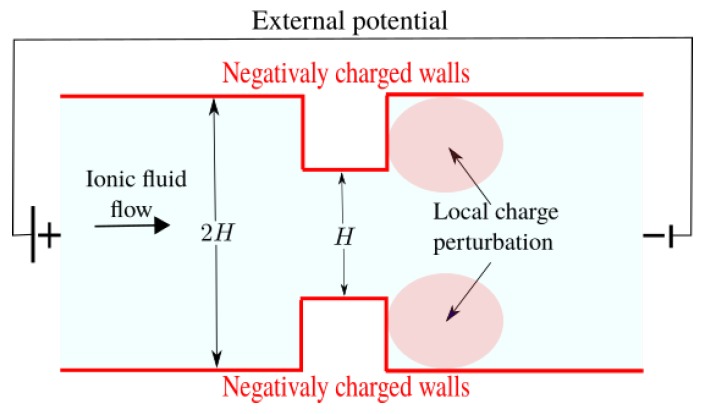
Illustration of imposed perturbation near the corners.

Channels with contractions and expansions are generally used when one is interested in mixing different fluids in a microchannel. Here the idea is trying to mimic the behavior of the fluid flow for this type of geometry, specifically regarding to the electrical effects near the corners. Physical description of these effects related to the movement of the charges near the corners is very complex, but we can try in some way to find a coherent approach linking the phenomenon to numerical simulation. As experimentally found by [40], near the corners there are fluctuations in zeta potential on the walls, causing a change on the velocity in that neighborhood. Here, initially the proposal is to put on an external perturbation on the potential, which we will consider to be due to punctual charges located in the nozzle near the corners, as show in Figure 14. Let us consider that the charges are very distant from each other, so we will be not taken into account the isolated interaction between them. In addition, we consider that the interaction of the perturbation charges with the part before the contraction of the channel is negligible, assuming that the concentration of ions in this place remains stable, i.e., the ionic depletion starts when the fluid arrives inside the contraction of the channel, in the region with intense gradient in Figure 14. It is known from the electromagnetism theory that the potential φ(x,y) due to an elementary point charge is given by

(20) $$\varphi \left(x,y\right)=\frac{Ke}{\sqrt{{\left(x-x_{0}\right)}^{2}+{\left(y-y_{0}\right)}^{2}}},$$

where $P(x_{0},y_{0})$ is the place of the point charge is located, *K* is the dielectric constant and *e* the elementary charge. In this way, the potential $\psi_{p}$ on the walls near the corners is the sum of reference zeta potential with perturbation potential, $\psi_{p}=\zeta_{0}+\varphi$, and one can be write:

(21) $$\psi_{p}=\zeta_{0}\left[1+\frac{\omega }{\sqrt{{\left(x-x_{0}\right)}^{2}+{\left(y-y_{0}\right)}^{2}}}\right],$$

where we defined $\omega =KeH\nabla \phi /\zeta_{0}^{2}$ as a constant which depends on external applied field ∇ϕ, i.e., increasing ∇ϕ, increases the electro-osmotic velocity and the perturbation effect on the standard potential ψ is consequently accentuated. On the other hand, increasing the distance from the wall, decreases the potential due the perturbation resulting $\psi_{p}\to \zeta_{0}$ from Equation (21). Additionally, the distance between parallel plates must be greater than the distance of action of the imposed perturbation, i.e., $\sqrt{{\left(x-x_{0}\right)}^{2}+{\left(y-y_{0}\right)}^{2}}\ll H$. By making $\psi_{p}=\psi_{p}^{*}\zeta_{0}$, $x=x^{*}H$ e $\omega =\omega^{*}H$, one can write the expression for dimensionless potential:

(22) $$\psi_{p}^{*}=1+\frac{\omega^{*}}{\sqrt{{\left(x^{*}-x_{0}^{*}\right)}^{2}+{\left(y^{*}-y_{0}^{*}\right)}^{2}}}.$$

According to [41], the potential on the corners have an inverse square root of ionic concentration dependence, $\psi_{p}∼n^{-1/2}$. In this way, the ionic concentration depends on inverse square of potential, lead to write the following relation:

(23) $$n_{p}=n_{bc}\frac{\zeta_{0}^{2}}{\psi_{p}^{2}},$$

where $n_{bc}$ is the value of ionic concentration on the channel wall without perturbation effect. When $\psi_{p}$ approaches the zeta potential, perturbation effects are negligible then the ionic concentration is given by $n_{p}=n_{bc}$. On the other hand, if $\psi_{p}$ increases due the perturbation effect, the ionic concentration decreases representing depletion of charges near the corners. Using the expression for $\psi_{p}$ one can writing Equation (23) in dimensionless form:

(24) $$n_{p}^{*}=\frac{n_{bc}^{*}}{{\left(1+\frac{\omega^{*}}{\sqrt{{\left(x^{*}-x_{0}^{*}\right)}^{2}+{\left(y^{*}-y_{0}^{*}\right)}^{2}}}\right)}^{2}}.$$

For this proposal, the Equations (22) and (24) were implemented on the boundaries near the corners. Results are obtained for different values of the perturbation. First the images of flow properties in the nozzle are showed.

**Figure 15:**
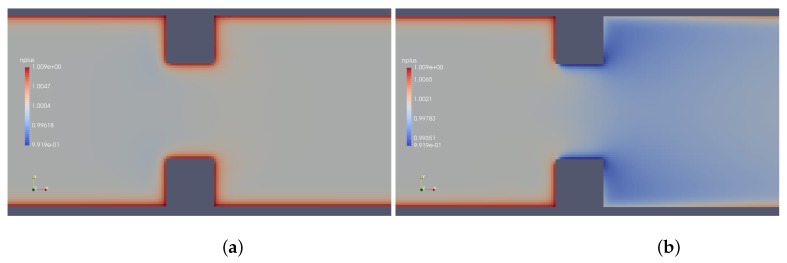
Ionic concentration $n^{+}$. (**a**) $\omega^{*}=0$ and (**b**) $\omega^{*}=6.0\times 10^{-3}$.

**Figure 16:**
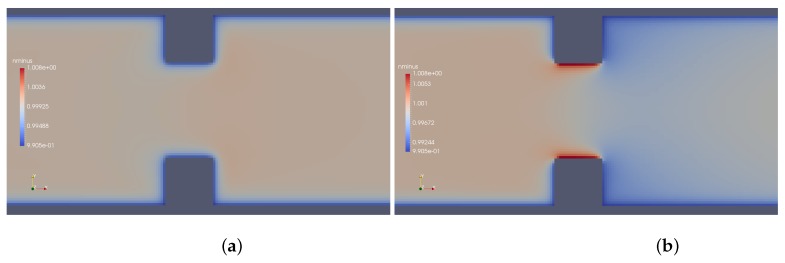
Ionic concentration $n^{-}$. (**a**) $\omega^{*}=0$ and (**b**) $\omega^{*}=6.0\times 10^{-3}$.

**Figure 17:**
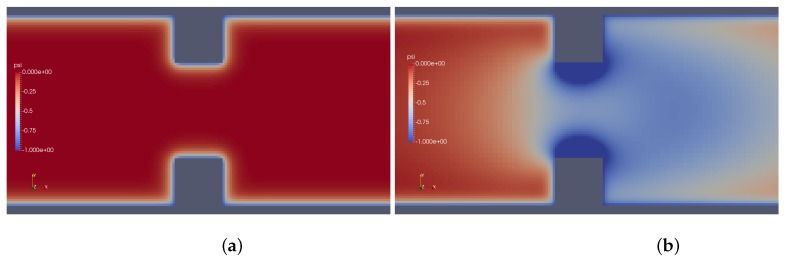
Potential ψ. (**a**) $\omega^{*}=0$ and (**b**) $\omega^{*}=6.0\times 10^{-3}$.

**Figure 18:**
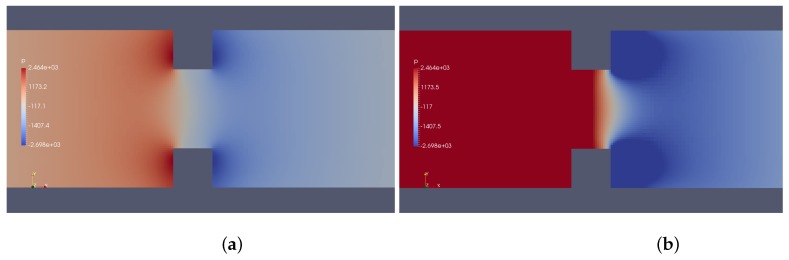
Pressure *p*. (**a**) $\omega^{*}=0$ and (**b**) $\omega^{*}=6.0\times 10^{-3}$.

**Figure 19:**
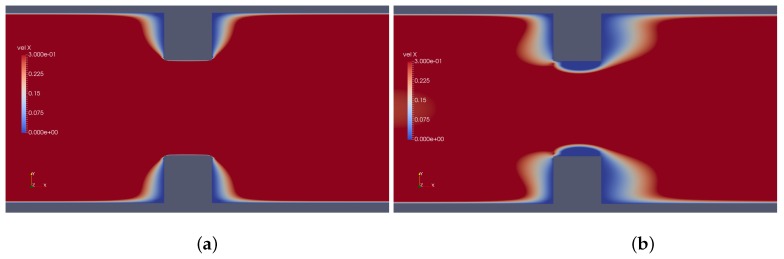
Downstream velocity, in (**a**) without perturbation, i.e., $\omega^{*}=0$ and (**b**) $\omega^{*}=1.0\times 10^{-3}$.

**Figure 20:**
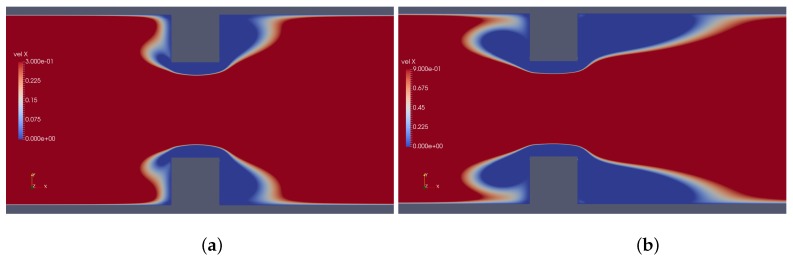
Downstream velocity, in (**a**) $\omega^{*}=3.0\times 10^{-3}$ and (**b**) $\omega^{*}=6.0\times 10^{-3}$.

Results provided by imposed perturbation are show in Figure 15, Figure 16, Figure 17, Figure 18, Figure 19 and Figure 20. Figure 15 and Figure 16 show the ionic concentrations $n^{+}$ and $n^{-}$ respectively, in (a) for zero perturbation $\omega^{*}=0$ and (b) for strong corner effect imposed by $\omega^{*}=6.0\times 10^{-3}$, where accentuated charge changes was observed after contraction in the channel. These changes are directly linked to the ψ changes and in fact occurs due to the coupling of Poisson–Nernst–Planck equations. The perturbation effect increases as $\omega^{*}$ is increased, as show in Figure 17. Therefore, the perturbation make changes on the pressure and velocity near the corners as show in Figure 18 and Figure 19. Increasing the perturbation parameter $\omega^{*}$, the flow is concentrate in the center of the channel as show in Figure 20, reducing the cross-section area of the fluid flow, contributing in the case of fluid mixing to be more efficiently process.

**Figure 21:**
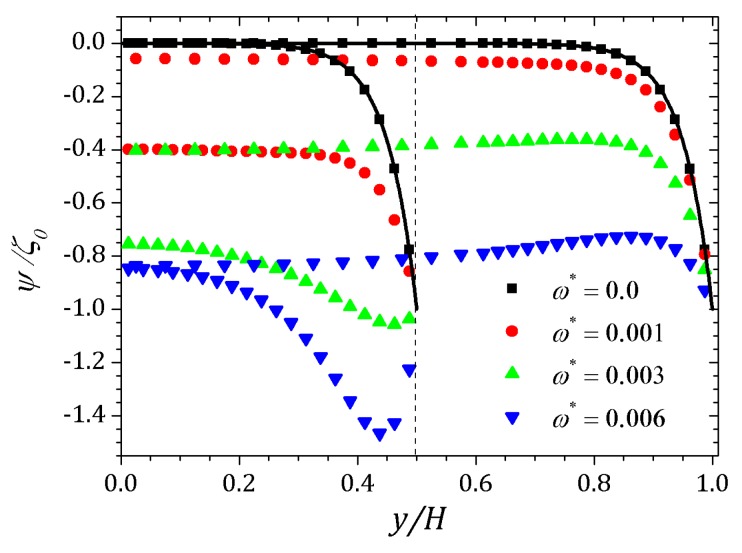
Potential ψ into the contraction and right after the expansion of the channel. Results for perturbation parameter $\omega^{*}=0$, $1.0\times 10^{-3}$, $3.0\times 10^{-3}$ and $6.0\times 10^{-3}$.

Figure 21 shows the curves of potential ψ as a function of the applied perturbation. For $\omega^{*}=0$, there is no perturbation and the potential curves represented by squares are similar to that curve obtained for the parallel plate channel. By imposing $\omega^{*}=1.0\times 10^{-3}$, curves represented by circles, noticed an increase in the absolute value of ψ in the center of the contraction and after the expansion, ψ approaches zero again indicating the decrease of perturbation effect out of the contraction. Increasing the perturbation parameter, $\omega^{*}=3.0\times 10^{-3}$, curves represented by upwards triangles, can be seen an more accentuated increment in the potential within the contraction and after the expansion of the channel. For $\omega^{*}=6.0\times 10^{-3}$, curves represented by downward triangles, perturbation effect changes the potential to have approximately the same value in the plateau within and after the contraction.

**Figure 22:**
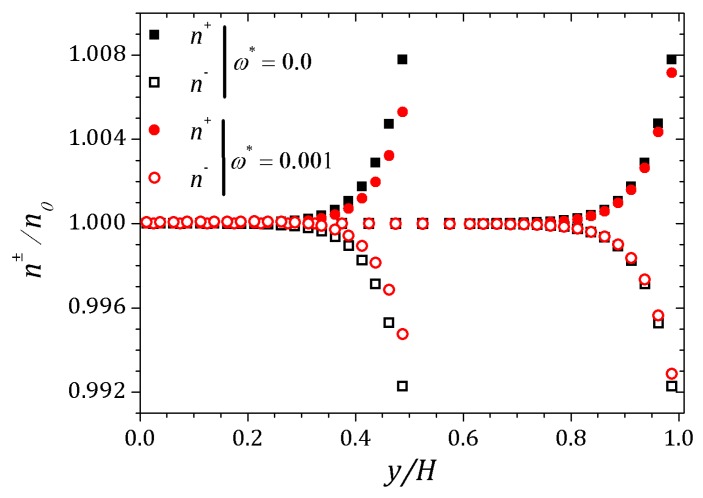
Ionic concentration $n^{\pm }$ into the contraction and right after the expansion of the channel. Results for perturbation parameter $\omega^{*}=0$ and $1.0\times 10^{-3}$.

**Figure 23:**
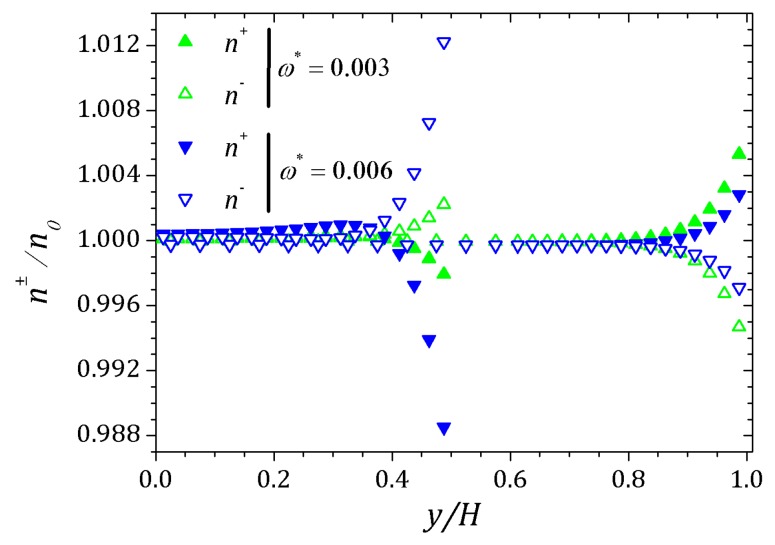
Ionic concentration $n^{\pm }$ into the contraction and right after the expansion of the channel. Results for perturbation parameter $\omega^{*}=3.0\times 10^{-3}$ and $6.0\times 10^{-3}$.

**Figure 24:**
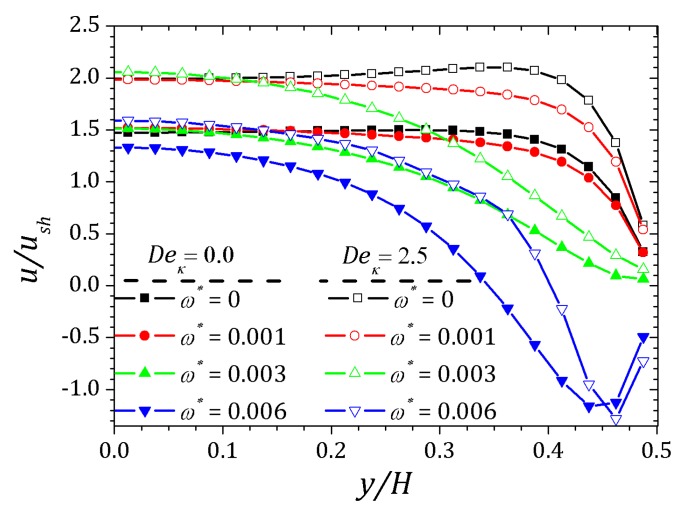
Perturbation effect on the velocity profile for sPTT model. Results for $De_{\kappa }=2.5$, ε=0.1 and perturbation parameter $\omega^{*}=0$, $1.0\times 10^{-3}$, $3.0\times 10^{-3}$ and $6.0\times 10^{-3}$.

**Figure 25:**
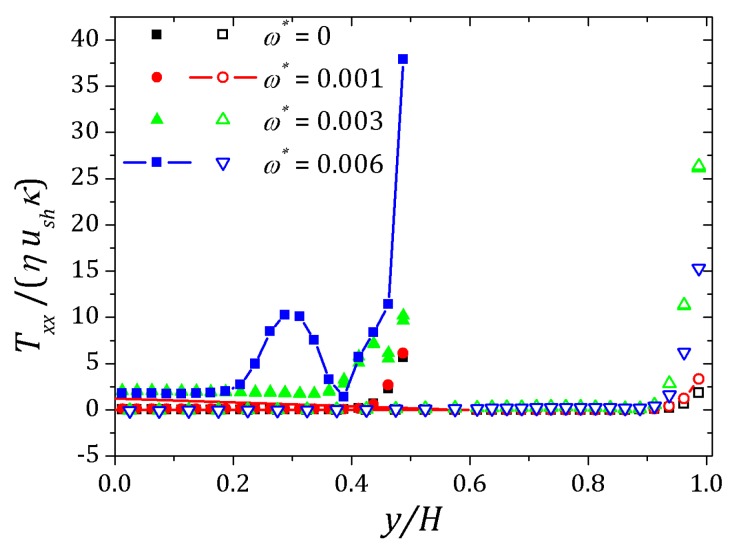
Normal stress into the contraction and right after the expansion of the channel. Results for $De_{\kappa }=2.5$, ε=0.1 and perturbation parameter $\omega^{*}=0$, $1.0\times 10^{-3}$, $3.0\times 10^{-3}$ and $6.0\times 10^{-3}$.

**Figure 26:**
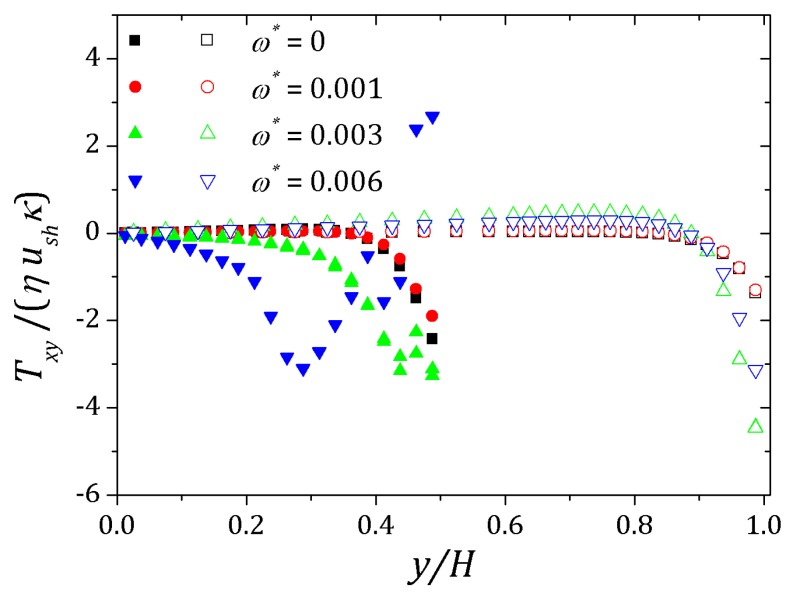
Shear stress into the contraction and right after the expansion of the channel. Results for $De_{\kappa }=2.5$, ε=0.1 and perturbation parameter $\omega^{*}=0$, $1.0\times 10^{-3}$, $3.0\times 10^{-3}$ and $6.0\times 10^{-3}$.

The behavior of the ionic concentration curves is shown in Figure 22. For $\omega^{*}=0$, curves represented by squares, the behavior is similar to that obtained for parallel plates channel. If $\omega^{*}=1.0\times 10^{-3}$, curves represented by circles, we observe a decrease in concentration $n^{+}$ and an increase of $n^{-}$ within the contraction and this effect is smaller after the channel expansion. When $\omega^{*}=3.0\times 10^{-3}$ the concentration $n^{+}$ decreases while $n^{-}$ increases and the curves are inverted relative to the reference concentration $n_{0}$, as shown in Figure 23. This effect is even more pronounced for $\omega^{*}=6.0\times 10^{-3}$. In this way, the coupling between the potential and ionic concentrations is related in Figure 21, Figure 22 and Figure 23, indicating that the ionic concentration variation is affected by the potential increment due to perturbation. It is natural to expect that the perturbation imposed on the nozzle will cause numerical instabilities in the ionic concentration and potential ψ, since we are forcing a new distribution of charges for the problem, but if the perturbation is small enough, the numerical method is stable. The velocity profiles are affected by both the perturbation and the variation of the pressure within the contraction in the channel. These curves were obtained at the center of the contraction and Figure 24 shows the Newtonian and viscoelastic profiles. The curves represented by squares correspond to $\omega^{*}=0$, i.e., velocity profile variations occur due to the variation of the pressure in the contraction, and tends to form a crest before to the velocity curve decay and then decreases until zero on the wall, where full squares correspond to the velocity for the Newtonian fluid. This effect is most accentuated for the viscoelastic fluid, as can be seen in the curve represented by empty squares. Increasing the perturbation effect, $\omega^{*}=1.0\times 10^{-3}$, the crests are suppressed as shown the curves represented by full and empty circles corresponding to the Newtonian and viscoelastic fluid, respectively. If $\omega^{*}=3.0\times 10^{-3}$ the profile forms a crest in the center of the channel and increases the distance from the center, the velocity tends to zero quickly, as shown the curves with upwards triangles. This effect is similar to that applying a pressure gradient on the channel ends, as seen in Section 4.2. Finally, if $\omega^{*}=6.0\times 10^{-3}$, it is observed the existence of negative velocities near the wall, represented by the downwards triangles. Behavior of the polymeric tensor, Figure 25 and Figure 26, corroborates with the results obtained in Section 4.2 in order to show the similar effect to of applying a negative pressure gradient when the fluid enters in the contraction, represented by the fully points and a positive pressure gradient after the expansion of the channel represented by the empty points.

## 5. Conclusions

Computational experiments were performed to obtain the solution of electro-osmotic flows. In order to validate the HiG-Flow, first the results for the pure electro-osmotic fluid flow of viscoelastic fluids (using the full PTT) through parallel plates were obtained and compared with satisfactory accuracy against analytical results. Later, the sPTT model was studied and it was seen the influence of an applied gradient pressure on the flow for a parallel plates channel. Furthermore, it was observed the vortex formation in a nozzle by imposed perturbation near the corners. Results were obtained and the contraction in the channel naturally imposes a pressure gradient on that neighborhood and then it was found that the imposed perturbation near the corners causes a similar effect. It is notable that the imposition of the perturbation causes instabilities on the solution, as can be seen in the concentration and potential curves. It is expected because one is imposing conditions unknown to the flow, but for weak perturbation the numerical solution keep stable.
